# Production of Marker-Free Apple Plants Expressing the Supersweet Protein Gene Driven by Plant Promoter

**DOI:** 10.3389/fpls.2019.00388

**Published:** 2019-03-29

**Authors:** Vadim Timerbaev, Tatiana Mitiouchkina, Alexander Pushin, Sergey Dolgov

**Affiliations:** ^1^Laboratory of Expression Systems and Modification of the Plant Genome “Biotron”, Branch of the Shemyakin-Ovchinnikov Institute of Bioorganic Chemistry, Russian Academy of Sciences, Pushchino, Russia; ^2^Laboratory of Plant Bioengineering, Nikita Botanical Gardens – National Scientific Center, Russian Academy of Sciences, Yalta, Russia; ^3^Laboratory of Plant Genetic Engineering, All-Russia Research Institute of Agricultural Biotechnology, Russian Academy of Sciences, Moscow, Russia

**Keywords:** *Agrobacterium*-mediated transformation, marker-free plants, *Malus* × *domestica*, thaumatin II, cytosine deaminase, R/RS recombination system

## Abstract

The presence of antibiotic resistance and other marker genes in genetically modified plants causes concern in society because of perceived risks for the environment and human health. The creation of transgenic plants that do not contain foreign genetic material, especially that of bacterial and viral origin, largely alleviates the tension and makes the plants potentially more attractive for consumers. To produce marker-free transgenic apple plants, we used the pMF1 vector, which combines *Zygosaccharomyces rouxii* recombinaseR and a *CodA-nptII* bifunctional selectable gene. The thaumatin II gene from the tropical plant *Thaumatococcus daniellii*, which is under the control of the plant E8 gene (a predominantly fruit-specific promoter) and rbsS3A terminator, was taken as the gene of interest for modification of the fruit taste and enhancing its sweetness. Exploitation of this gene in our laboratory has allowed enhancing the sweetness, as well as improving the taste characteristics, of fruits and vegetables of plants such as strawberry, carrot, tomato and pear. We have obtained three independent transgenic apple lines that have been analyzed by PCR and Southern blot analyses for the presence of T-DNA sequences. Two of them contained a partial sequence of the T-DNA. With one line containing the full insert we then used a delayed strategy for the selection of marker-free plants. After induction of recombinase activity in leaf explants on selective media with 5-fluorocytosine (5-FC) we obtained more than 30 sublines, most of which lost their resistance to kanamycin. Most of the apple sublines showed the expression of the supersweet protein gene in a wide range of levels as detected by RNA accumulation. The plants from the group with the highest transcript level were propagated and grafted onto dwarf rootstocks for early fruit production for future estimates of protein levels and organoleptic analyses. Thus, we developed a protocol that allowed the production of marker-free apple plants expressing the supersweet protein.

## Introduction

Despite the increase in the number of genetically engineered forms of plants and their evident economic expedience, such crops are quite cautiously accepted by society, primarily due to the presence of foreign genetic material from distant organisms (bacteria, viruses, etc.) for the expression of target genes in transgenic plants and as selective markers of resistance to antibiotics and herbicides. Under these conditions special urgency is given to techniques by which new highly productive forms of crops can be created without foreign genetic material, primarily of bacterial origin, and without genes of antibiotic resistance, the lack of which is probably to facilitate the release genetically modified organisms into open systems especially in regions with distrust related to GMO such as the European Union. For example, in 2010 Amflora potato, which has high amylopectin content, was approved for commercial cultivation in the EU. Amflora was criticized for the presence of the antibiotic resistance *nptII* gene. In 2011, BASF has stopped selling its Amflora potatoes in the EU due to widespread public and political resistance and finally variety was withdrawn from the EU market by the European Commission in 2013 due to procedural errors during the approval process (Kamle et al., 2017). The European Commission has stated in one of the report that cisgenic crops are becoming more accepted in Europe (Gaskell et al., 2013). To meet modern trends, some governments are implementing global projects with approaches to minimize foreign DNA in the genomes of commercial crops. From 2006 to 2015 Wageningen University and Research Centre carried out a project on Durable Resistance in potato against *Phytophthora* aimed at stimulating research on genetic modification and public debate on innovative genetic techniques like cisgenesis (Haverkort et al., 2016).

In recent years the use of alternative strategies that allow transformed cells to obtain an advantage (for example, the marker gene encodes an enzyme that converts a selective agent into the metabolically available form, or usage of alternative nutrient sources Sidorova et al., 2017) is gaining in popularity. Such systems are referred to as positive selection. In some cases, the use of positive selection can significantly improve the transformation frequency, but its main advantage lies in the absence of antibiotic resistance genes in resulting plants. The strategies for marker gene elimination from the nuclear genome are a fundamentally different approach. They include co-transformation of the transgenes with the subsequent segregation (Daley et al., 1998), removal of marker genes by means of transposons (Goldsbrough et al., 1993) and strategies based on site-specific recombination to allow the elimination of undesired DNA after appropriate treatment (Gleave et al., 1999). To date, three systems based on site-specific recombinase are well studied and described. These are the Cre/lox system of the P1 phage (Dale and Ow, 1991), the FLP/FRT recombinase system from *Saccharomyces cerevisiae* (Lyznik et al., 1996) and the R/RS recombinase system from *Zygosaccharomyces rouxii* (Onouchi et al., 1995; Sugita et al., 2000). One of the examples of marker-free technology development may be multi-auto-transformation vectors (MAT) (Ebinuma et al., 1997). The first systems were based on the visual selection of transgenic plants containing *ipt* or *rol* oncogenes (Ebinuma et al., 1997; Ebinuma and Komamine, 2001). Thus, the use of oncogenes as selective agents allows easy visual selection of transformed tissues; however, obtaining transgenic plants with a normal phenotype requires the removal of the oncogenes from the genome. In the first MAT systems, the deletion of these genes was carried out using inverted repeats of the transposon *Ac* from maize; the frequency of loss of the *ipt* marker gene was very low, however (0.1–0.5%). Later, the R/RS recombination system began to be used to remove the marker gene (Sugita et al., 1999, 2000), whereby the removal efficiency has been improved significantly, but initially the elimination of foreign sequences occurred spontaneously in MAT systems due to the constitutive expression of recombinase, which made the process uncontrollable, and led to unpredictable results. An additional increase in the frequency of marker gene-free transgenic lines was achieved by the use of a dexamethasone-inducible expression system to remove the marker gene *ipt* (Kunkel et al., 1999). After selection of the transformants, the chemical activation of recombinase leads to elimination of the unnecessary portion of T-DNA from a plant genome. Despite the wide use of these vectors to produce transgenic plants of different species, not very many studies have been devoted to obtaining marker-free plants with beneficial features.

Since the methods of obtaining marker-free organisms have acquired special relevance only in the last decade, most of the studies are devoted to the development of new technologies and vector systems. In this work, to remove the undesirable DNA we used a site-specific recombinase belonging to the pMF vectors (Plant Research International, Wageningen, Netherlands) (Schaart et al., 2004). The main advantage of this system is its sequential double selection. The first stage after *Agrobacterium*-mediated transformation is the selection of regenerants by using antibiotics such as kanamycin, hygromycin, or phosphinotricine, and further elimination of DNA sequences, flanked by the intact recombination sites (RS) occurs as a result of the chemical activation of recombinase R that is inactivated in the presence of the ligand-binding domain (LBD) of a glucocorticoid receptor. Its activation is carried out after incubation of the plant tissue in a solution of dexamethasone (Dex). Further selection on a medium with 5-fluorocytosine (5-FC) prevents the development of plant tissues containing the *codA* gene (cytosine deaminase converts non-toxic 5-FC to cytotoxic 5-fluorouracil); it thus becomes impossible to obtain chimeras due to incomplete elimination of the DNA. This system was successfully applied in cultures such as strawberries, apples, and pears (Schaart et al., 2004; Vanblaere et al., 2011; Righetti et al., 2014; Krens et al., 2015).

Based on pMF1 vector we created a plasmid containing the gene for supersweet thaumatin II protein under the control of the fruit-specific E8 gene promoter and rbsS3A gene terminator. Thaumatin II was first isolated from the fruits of the tropical plant katemfe (van der Wel and Loeve, 1972); it is 3000 times sweeter than sucrose (Nikoleli and Nikolelis, 2012). The thaumatin threshold value of sweetness in humans is 50 nM, one of the lowest of any sweet-tasting protein. The perception of sweetness is delayed to some extent and it leaves a slight liquorice-like after-taste (Nairn et al., 1986). Moreover, thaumatin II has an anti-fungal activity (Vigers et al., 1992; Popowich et al., 2007), so in addition to improving the taste of fruits of agricultural crops the heterologous expression of the thaumatin II gene makes it possible to increase the resistance of these plants to fungal phytopathogens. In the first studies with the thaumatin II gene, potatoes (Witty, 1990) and cucumber (Szwacka et al., 1996) plants were used as objects. Expression of the thaumatin II gene in transgenic cucumber causes not only a sweeter taste, but also higher aroma acceptability (Szwacka et al., 1996). Presently, a wide range of cultures expressing this supersweet protein has been obtained including strawberries, carrots, pears, and others (Schestibratov and Dolgov, 2005; Dolgov et al., 2011; see reviews by Szwacka et al., 2012 and Firsov et al., 2018). In the fruits of these plants a recombinant protein was detected and a modification of the taste was observed increasing their sweetness and attractiveness. Increasing the sweetness of apple fruit is an actual problem in temperate climates, like in Russia. In the cultures listed above, control of thaumatin II gene expression is controlled by the 35S virus promoter and the plant genome contains selective genes. For breeding purposes it is desirable to produce plants that lack the antibiotic resistance genes and display fruit-specific expression of the thaumatin II gene. For this, we used one of the well-characterized promoters of the tomato E8 gene. Transcription of E8 gene is activated at the onset of fruit ripening (Deikman et al., 1992), so promoter of E8 gene reported as fruit-ripening specific.

The objective of this study was to obtain marker-free apple plants with the gene for supersweet protein thaumatin II under control of plant fruit-specific promoter and conduct analysis of its expression in the transgenic plants.

## Materials and Methods

### Creation of a Binary Vector

The binary plasmid pMF-E8 is based on pMF1 (Schaart et al., 2004) was used for the *Agrobacterium*-mediated transformation of apple plants. The coding sequence of thaumatin II gene was obtained by PCR from plasmid pUR528 containing a fragment of preprothaumatin II (Edens et al., 1982) using primers Thau-CDS (Table 1). An 1195 bp fragment of tomato fruit-specific promoter E8 (GenBank accession No. AF515784.1) and a 402 bp fragment of tomato *rbcS* gene terminator (GenBank No. X05984.1) were obtained by PCR from the genomic DNA of tomato cv. Yalf with the sets of oligonucleotides E8-prom and 3A-ter, respectively. Amplified PCR products were sequenced by Evrogen (Moscow, Russia) to verify the gene identity and cloned into the modified pUC18. The resulting expression cassette then was placed into the pMF1 vector using AscI and SbfI. DNA cloning was performed according to standard procedures (Maniatis et al., 1982).

**Table 1:** Primer sequences and amplicon sizes.

Acronym	Gene or combination	Forward/reverse primer sequences (5′–3′)	Amplicon size (bp)	Method
E8-prom	E8 (promoter)	CTATCCCGGGAGGCGCGCCAGAAGGAATTTCACGAAATC/TCAGGATCCCTTCTTTTGCACTGTGAATG	1146	PCR (cloning)
3A-ter	Ribulose-1,5-bisphosphate carboxylase/oxygenase (*rbcS3A*) terminator	ACTGACCGGTTCTAGAAAAACTAATTGCC/ACTCCTGCAGGCGAGGGAGTAGTAGAGATAAG	423	—»—
Thau-CDS	Thaumatin II (CDS)	TAGGATCCATGGCCGCCACCAC/ACTACCGGTTTACTCGTCTTCAAGTTCAAG	725	PCR (cloning), Southern blotting
(1) RS site	RS site-35S promoter	CGATTTGATGAAAGAATGAATTAATG/GTGTGTCGTGCTCCACCATG	526	PCR
(2) *CodA*	CaMV35S promoter- Cytosine deaminase	CCAACCACGTCTTCAAAGCA/AATGCCTTCAAACAGCGTGC	589	—»—
(3) *NptII*	Neomycin phosphotransferase II	TCTGATGCCGCCGTGTTCC/ATGCGCGCCTTGAGCCTG	440	PCR, RT-PCR, Southern blotting
(4) Nos ter	Nopaline synthase terminator	CCGATCGTTCAAACAT/GTAACATAGATGACACCGCG	249	PCR
(5) 35S prom	CaMV35S promoter	AGCACGACACACTTGTCTACTC/CTCTCCAAATGAAAT	406	PCR
(6) *RecR*	Recombinase R	ATGCGCAAGGAGGCAGGTCG/GCCACACGGGAGACGCCTTC	637	PCR, RT-PCR, Southern blotting
(7) *Thau*	Thaumatin II	GCGCTGCCACCTTCGAGATCG/GCAGGTGACGGTGGTTGGCT	584	PCR, RT-PCR
(8) E8-Thau	E8 promoter-thaumatin II	CTTAATCAGACGTATTGGGTTTC/AGCCTTTGATGTTGGAGATGTC	624	PCR
(9) Thau-ter	Thaumatin II-rbcS3A terminator	GCTCAACCAGTACGGCAAGG/CAAGGGAAAACCCAAAGGAG	445	—»—
Actin	Actin 11	TCATCATACTCGGCCTTCGC/CCATCCATGATTGGAATGGAAGC	302	RT-PCR
*THFS*	Formate-tetrahydrofolate ligase	AGCAGCGTTGAATACTCAGAG/ATACTGGGTTTTCGCCATGC	99	Real-time PCR
*TMp1*	Type 1 membrane protein-like	AGACCGACTCAATGTTGCTCTC/GTGGAAGGTGGTGCAAATCC	73	—»—
*ACT11*	Actin 11	GCTGTTCTTTCCCTCTACGC/GCATGGGGAAGAGCATATCC	110	—»—
*Th84*	Thaumatin II	AGAGTCCTGGACCATCAAC/CCGCTGTCGTCGAAATAG	84	—»—
*Th113*	—»—	GCACCGTGTTCCAGACGAG/GTCCAGGACATAACTGAACGC	113	—»—
Th95	—»—	AGCTCAACTCGGGAGAGTC/GCTGTCGTCGAAATAGCAGTC	95	—»—


### Apple Transformation and Marker-Free Plant Production

Apple plants of a hybrid obtained from free pollination of the Melba variety from Central Genetic Laboratory (Michurinsk, Russia) were used in our experiments. This hybrid has the best characteristics of the parent variety while at the same time having an increased resistance to low temperatures, which is important for the temperate climate conditions.

The plants were cultivated *in vitro* on QL medium (Quorin and Lepoivre, 1977) containing 1.5–2.0 mg L^-1^ 6-benzylaminopurine (BAP) and 0.3 mg L^-1^ indole-3-butyric acid (IBA), and rooted on half-strength QL medium supplemented with 0.5–1.0 mg L^-1^ IBA. Leaf pieces from *in vitro*-rooted plants were used for transformation. Co-cultivation of leaf explants with *Agrobacterium tumefaciens* strain AGL0 (Hood et al., 1986) carrying plasmid pMF-E8 was performed on MS medium (Murashige and Skoog, 1962) containing 3.0 mg L^-1^ *N*-(2-chloro-4-pyridyl)-*N*-phenylurea (4CPU), 1.0 mg L^-1^ 1-naphthaleneacetic acid (NAA), and 1 mg L^-1^ 2,3,5-triiodobenzoic acid (TIBA) for 2–3 days. Regeneration was performed in the dark on the same medium supplemented with 500 mg L^-1^ cefotaxime and 35 mg L^-1^ kanamycin. Apple shoots were continuously propagated and rooted on half-strength QL medium with the addition of 0.5 mg L^-1^ IBA and 25 mg L^-1^ kanamycin. Analyzed plants of transgenic lines 1 and 6 were subcultured on propagation medium and leaf explants from these shoots were incubated overnight on agarised MS medium supplemented with 10 μM Dex and then transferred to propagation medium containing 2 μM Dex and 250 mg L^-1^ 5-FC to obtain regenerants again. Putative marker-free plants obtained were then rooted and propagated on shoot propagation medium containing 250 mg L^-1^ 5-FC and were moved to the greenhouse for further analysis.

### Southern Blot Analysis of Putative Marker-Free Plants

**FIGURE 1:**
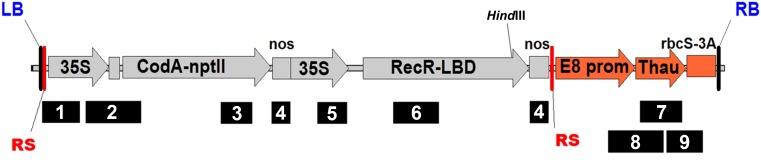
Schematic representation of the pMF-E8 vector T-DNA region with primer set positions. RS, recombination site; RB and LB, right and left border of T-DNA, respectively; 35S, CaMV35S promoter; *CodA-nptII*, translational fusion gene for positive (*nptII*) and negative (*codA*) selection; nos, nopaline synthase terminator; Rec-LBD, translational fusion of Recombinase R-LBD; E8 prom, E8 promoter; Thau, thaumatin II; rbcS3A, rbcS3A terminator. For interpretation of primer set numbers, see Table 1. *HindIII*, position of the restriction site for which the DNA was digested for the Southern blot assay.

Apple genomic DNA (30 μg) was digested overnight at 37°C with 60U *HindIII* (see position on Figure 1). The fragments were separated on 0.9% agarose gel and transferred to a positive-charged nylon membrane Hybond N+ (GE Healthcare, Little Chalfont, United Kingdom) by capillary blotting following the manufacturer’s instructions. The DNA probes were constructed by PCR using plasmid pMF-E8 as the template. DNA probes of 725 bp (for thaumatin II gene), 440 bp (for *nptII* gene) or 637 bp (for *RecR* gene) were labeled with alkaline phosphatase using Amersham Gene Images AlkPhos Direct Labelling and Detection System (GE Healthcare). Prehybridization, hybridization (overnight at 62°C) with alkaline phosphatase-labeled probes, and subsequent washings of the membrane were carried out according to the AlkPhos Direct Labeling System protocol. Detection was performed using CDP-Star detection reagent following the manufacturer’s directions (Amersham CDP-Star Detection reagent, GE Healthcare). The signal from the blot was accumulated on X-ray film (Retina XBE blue sensitive, Carestream Health INC., NY, United States) in film cassette at room temperature for 24 h. X-ray films were scanned on Amersham imager 600 (GE Healthcare Life Sciences, Japan) after development. The same blot was reprobed several times (ThauII, nptII, and RecR). The blot was striped in a 0.5% (w/v) SDS solution at 60°C for 60 min as described in the protocol for Amersham Gene Images AlkPhos Direct Labelling and Detection System.

### Nucleic Acid Extractions

The total DNA was isolated from young leaves of *in vitro* apple plants according to the method described by Rogers and Bendich (1994).

RNA samples were extracted from leaves of *in vitro* transgenic and control plants using GeneJET Plant RNA Purification Mini Kit (Thermo Fisher Scientific, Waltham, MA, United States). Each sample was treated with DNAse (Thermo).

### PCR Experiments

Primary apple transformants and all selected on medium with 5-FC regenerants were studied in detail for the presence of target and selective genes and their regulatory elements by PCR analysis. Nine pairs of primers were used for PCR analysis of the putative marker-free plants (Table 1). Reactions were carried out using HS Taq polymerase (Evrogen) as follows: 1 cycle of 5 min at 95°C, followed by 29 cycles of denaturation at 94°C for 20 s, annealing 20 s, extension at 72°C for 40 s, and one final cycle of 3 min at 72°C. PCR products were separated using 1.2% agarose gel electrophoresis, visualized with ethidium bromide under UV light and photographed.

### Semiquantitative RT-PCR and Real-Time qPCR

The cDNA was synthesized using Maxima H Minus Reverse Transcriptase (Thermo) according to the manufacturer’s protocol using oligo(dT)18 and random hexameter primers. For each sample, 3 μg of total RNA were taken for the reverse transcription reaction in a total volume of 20 μl following two-fold dilution. 2 μl of cDNA mix was used for PCR reaction. Housekeeping actin (Actin 11) gene as internal control was used for data normalization. PCR was performed using primer sets for recombinase R (*RecR*), neomycin phosphotransferase (*NptII*), and the thaumatin II (*Thau*) gene (Table 1) as described above. PCR cycles were chosen so that none of analyzed samples reached a plateau at the end of the amplification, that is they were in the exponential phase of amplification. The cDNA samples were checked for genomic DNA contamination by using controls without reverse transcriptase. The PCR products were separated in a 1.2% agarose gel, photographed and analyzed with the program GelQuant.NET^1^ for semi-quantitative estimation.

To quantify the RNA levels of the thaumatin II gene by quantitative real-time PCR, the cDNA of the control and transgenic apple plants obtained as described above were used. Three reference housekeeping genes were tested as endogenous control: *THFS*, *TMp1*, and *ACT11* (Perini et al., 2014; Table 1). We also designed three sets of primers for the target sequence of the thaumatin II gene (Table 1). For each set of primers the optimum annealing temperature was found using temperature gradients. We tested amplification efficiency using a series of 10-fold dilutions in triplicate. These values were used to calculate the standard curves and slopes and to compare the PCR efficiency. The experiments were performed on a LightCycler 96 instrument (Roche Diagnostics GmbH, Mannheim, Germany) using the PowerUp SYBR Green Master Mix reagent (Thermo) in plates (Axygen Biosciences, Union City, NJ, United States) in a volume of 20 μl. The PCR reaction conditions and the melting analysis of the products were selected according to the manufacturer’s recommendations. The 2^-ΔΔ^*^CT^* method (Livak and Schmittgen, 2001) was used to normalize and calibrate the *ThauII* values relative to the endogenous controls. Every sample was analyzed in triplicate. Data calculation and statistical analysis were performed using both the software supplied with the Roche device and Microsoft Excel. Primer sequences and amplicon sizes are given in Table 1.

## Results

### Construction of the Binary Vector

For obtaining the marker-free transgenic apple plants we used the vector pMF (Plant Research International, Wageningen) containing recombinase R from the yeast *Z. rouxii* fused to the LBD of the glucocorticoid receptor and the bifunctional selection gene *CodA-nptII*, allowing the selection of plants by negative selection on 5-FC after removal of the undesirable region of DNA from the genome. We have used the thaumatin II protein gene from the tropical plant *Thaumatococcus daniellii* cloned by Edens et al. (1982) as the sense gene under the control of tomato E8 gene fruit-specific promoter and tomato *rbcS* gene terminator. This protein has a supersweet flavor and exhibits antifungal activity, thus the expression of the gene allows improving the taste quality of fruits and increasing the resistance to phytopathogens. The resulting vector was named pMF-E8 (Figure 1).

### Apple Transformation and PCR Analysis of Plants

We carried out two transformation experiments in which 160 and 85 leaf explants were taken, respectively. Three kanamycin-resistant lines (two and one, respectively) were obtained as a result of *Agrobacterium*-mediated transformation of apple by using pMF-E8 vector. Transformation frequency was 1.2%. These plants were thoroughly tested by PCR for the presence of all genes from the T-DNA region and for the presence of both RS, which is a necessary condition for correct DNA excision. Results of the PCR analysis are shown in Table 2.

**Table 2:** Analysis of transgenic apple plants.

Line	RS site	*CodA*	*NptII*	*RecR*	35S prom.	Thau-CDS	Thau-ter
1	-	+	+	+	+	+	+
2	-	+	+	+	+	+	+
6	+	+	+	+	+	+	+


Insertion of the target thaumatin gene, as well as other genes of the transferred region, was detected in all three lines, whereas the presence of the recombination site, which is located closer to the left border of T-DNA, occurred only in line 6.

### Obtaining Marker-Free Apple Plants

The strategy of delayed selection was used to create marker-free apple plants. This strategy involves obtaining transgenic plants and their subsequent manipulation to remove the marker gene.

The following variants are presented in Table 3 were designed for experiments on obtaining the marker-free lines through the induction of recombinase and the subsequent cultivation of explants on the medium with dexamethasone.

**Table 3:** Marker-free apple obtaining experimental options.

Code	Name	Induction, 10 μM Dex	Selection, 250 mg L^-1^ 5-FC (immediate)	Selection, 250 mg L^-1^ 5-FC (delayed for 7 days)	Intent
0-0	Without selection	-	-	-	Background regeneration
0-2	Delayed selection	+	-	+	DNA excision, selection begins after 7 days
250-0	Without induction	-	+	+	Background DNA excision
250-2	Immediate selection	+	+	+	DNA excision; impact of 5-FC and Dex on regeneration


**FIGURE 2:**
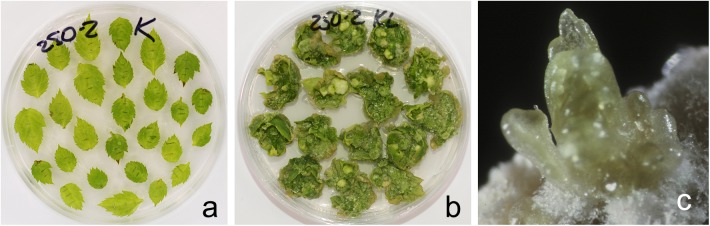
Obtaining marker-free apple plants. **(a)** Leaf explants of primary transgenic line 6. **(b)** Regeneration on medium supplemented with 5-FC. **(c)** Developing young shoot.

By using PCR it was found that the entire sequence of the T-DNA with the site of recombination at the left border is present only in line 6. The presence of both RS flanking the selective genes in the T-DNA region is a prerequisite for successful DNA removal. Nevertheless, we arbitrarily chose one of the remaining lines (line 1) for the first experiment as a negative control and line 2 was excluded. Two experiments were carried out; in the second experiment only the explants of line 6 were used, and variant 0-2 with delayed selection was excluded. From a total of 1800 apple leaf explants 45 regenerants were selected and propagated on the selective medium in the presence of 250 mg L^-1^ 5-FC. As a result of these experiments, five regenerants were obtained from explants of line 1 and 40 from explants of line 6 (Figure 2a–c and Table 4). Spontaneous recombination and DNA excision without treatment with Dex occured – four sublines were derived from explants of line 6.

**Table 4:** Selection of putative marker-free regenerants.

Event	Variant	Line	Number of explants		Number of regenerants		Regeneration efficiency, %	
					
			Initially^1^	Before regeneration^2^	Cut^3^	Rooted^4^	Primary^5^	Final^6^
I	0-2	1	127	101	8	1	7.9	1
		6	226	198	35	11	17.7	5.6
	250-0	1	159	159	2	0	1.3	0
		6	161	161	32	2	19.9	1.2
	250-2	1	155	152	27	4	17.8	2.6
		6	180	162	48	13	29.6	8
	0-0	NT	159	104	78	–	75	–
	250-2		142	119	66	–	55.5	–
II	250-0	6	253	53	12	2	22.6	3.8
	250-2		98	74	17	12	23.0	16.2
	0-0	NT	66	31	14	–	45.2	–
	250-2		75	37	10	–	27.0	–
Total number of obtained regenerants											45		


*^*1*^total number of cut explants; ^*2*^remaining number of explants after two passages; ^*3*^number of shoots separated from the explant for rooting; ^*4*^number of rooted shoots; ^*5*^ratio of cut shoots to the total number of explants before regeneration; ^*6*^ratio of rooted shoots to the total number of explants before regeneration.*

The strategy of immediate selection (250-2) was somewhat more effective compared to the delay for 7 days (0-2). The efficiency for line 6 was 8% for the first experiment and 16.2% in the second experiment and for the delayed selection the efficiency was 5.6%. Line 1 had an efficiency of 1% with the delayed and 2.6% with the immediate selection, suggesting either escapes or a low level of spontaneous deletions as line 1 did not have both recombinase sites. To test the negative effects of the selection and induction on regeneration, non-transformed controls were tested resulting in 55.5% regeneration on the selection and induction to 75% with no selection or induction in the first experiment and 27% relative to 45.2% in the second experiment.

### PCR and Southern Blot Analysis of Marker-Free Plants

**FIGURE 3:**
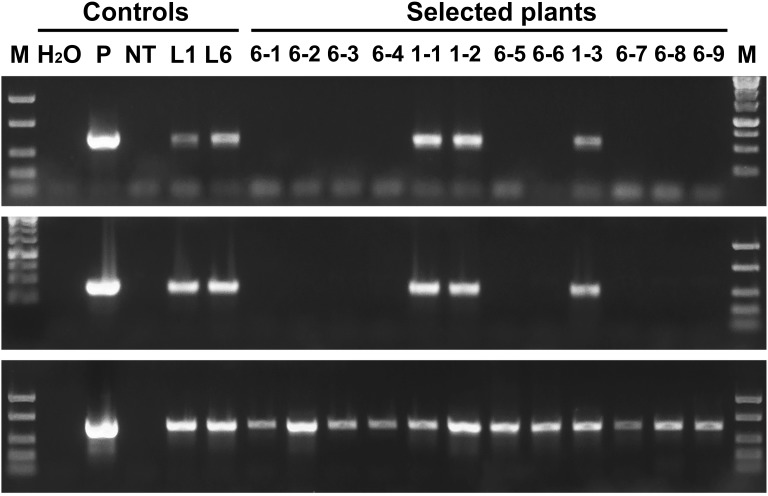
Molecular analysis of apple plants after recombinase induction. Polymerase chain reaction analysis using specific primers for *codA* (upper panel), *nptII* (middle panel) and *thauII* (bottom panel). M, molecular weight marker; H_2_O, water; P, plasmid pMF-E8; NT, non-transgenic plant; L1 and L6, primary transgenic lines; other numbers, obtained sublines, the first number indicates from which line the plant was derived.

**FIGURE 4:**
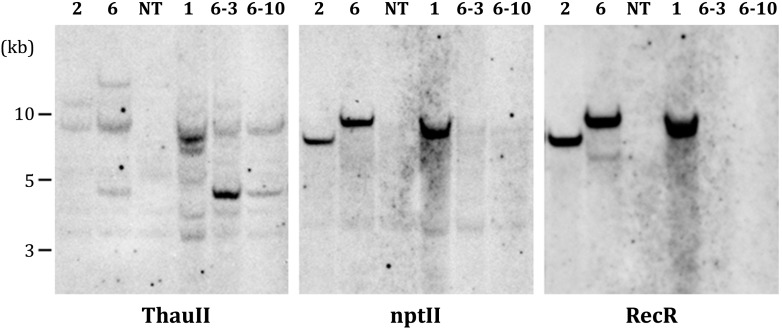
Southern blot analysis of parental (1, 2, and 6) and marker-free (6-3, 6-10) apple plants. NT, non-transgenic control.

PCR analysis revealed that all five sublines obtained from explants of line 1 are escapes, because in addition to the *thauII* gene they contain sequences of *nptII*, *CodA*, and *RecR* genes. There is no positive signal for selective genes in the PCR with genomic DNA of all sublines derived from line 6, while the gene of interest with its regulatory sequences is present in all forty sublines, which indicates more or less correct excision of both fusions of selective genes. The Figure 3 represents PCR analysis of 9 sublines derived from line 6 and 3 sublines derived from line 1 (reaction to the RecR gene is not shown). To confirm the results obtained by PCR, we performed Southern blot hybridization using genomic DNA of three primary and two potential marker-free sublines (Figure 4). It was found that the genomes of lines 1, 2, and 6 contain 5, 2, and 3 copies of the target gene, respectively, and one copy of each of the selective genes. Apparently there had been a partial integration of T-DNA region with the target gene only. After recombinase activation, probes for the *RecR* and *nptII* genes do not hybridize in both analyzed sublines, but two bands are detected for the thaumatin gene.

### Analysis of the Selective and Target Genes Expression by RT-PCR

**FIGURE 5:**
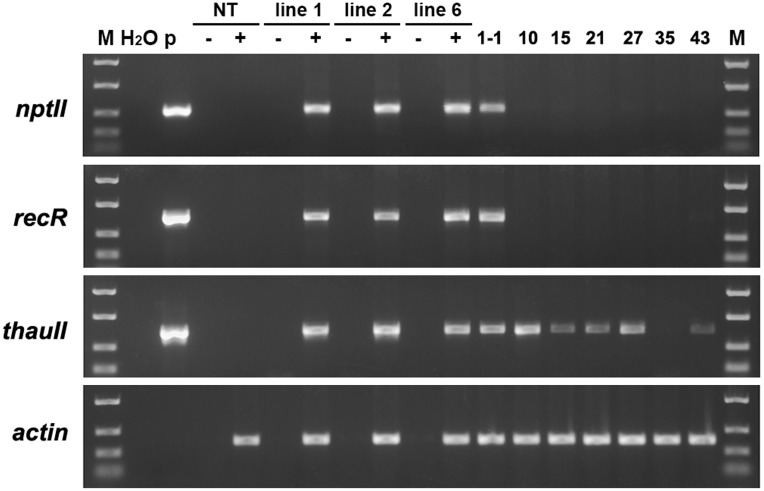
Semiquantitative RT-PCR analysis of four genes in apple plants. M, molecular weight marker; H_2_O, water; p, plasmid pMF-E8; NT, non-transgenic control; lines 1, 2, and 6, parent transgenic lines; other numbers, some of the produced sublines; –, reaction performed without the addition of reverse transcriptase; +, reaction performed with RT.

To estimate the transcription level of the target gene in marker-free apple plants we used semi-quantitative reverse transcription PCR. For this, the total RNA was isolated from young sterile plants, and then used as a matrix for reverse transcription. In the first experiment, in addition to the target gene, we analyzed the expression of selective genes of neomycin phosphotransferase (*nptII*) and recombinase (*RecR*) in the parental transgenic plants and some of the obtained sublines. The results of the analysis are shown in Figure 5. As confirmation of the alignment of produced cDNA, the product of the “housekeeping” gene actin was used. Expression of all genes from T-DNA was detected in three parental lines. In the mRNA of sublines derived from line 6 only the thaumatin II gene remained after excising undesired DNA. In subline 1-1, obtained from line 1, mRNA of selective genes was also detected. Previously, using PCR and Southern blotting, it was found that the plant of this subline is an escape that has passed through the stage of negative selection.

**FIGURE 6:**
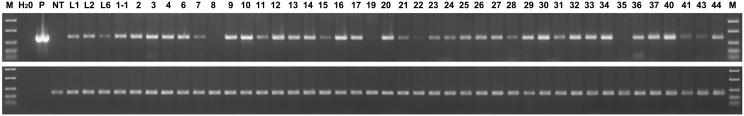
Semiquantitative RT-PCR analysis of thaumatin II gene expression in marker-free apple plants. In the upper panel the thaumatin gene is shown, in the lower panel the housekeeping actin gene. M, molecular weight marker; H_2_O, water; p, plasmid pMF-E8 (used for plant transformation); NT, non-transgenic control; L1, L2, and L6, parent transgenic lines; other numbers, marker-free sublines (except 1-1).

Further, using semi-quantitative RT-PCR analysis, we estimated the relative level of expression of the thaumatin II gene in all the resulting marker-free apple plants (Figure 6). The actin gene shows that the amount of cDNA taken for the reaction is the same in all analyzed samples. The difference in signal level of the target gene indicates individual characteristics of the apple sublines. Despite the presence of a target gene sequence in all apple plants, the expression of the thaumatin II gene in these experimental conditions could not be detected in two sublines and in another six sublines it was very weak. Using the GelQuant program, the relative level of expression was compared among all the resulting marker-free sublines and eight selected super-expressing plants were identified.

### Analysis of the Target Gene Expression by Real-Time PCR

Before the experiments on the evaluation of the level of expression of the transferred thaumatin II gene in apple plants, it was necessary to determine whether the selected primers are suitable for real-time PCR quantification and their specificity, and also to determine the optimum annealing temperatures for each pair. For this, PCR reactions were carried out with an annealing temperature gradient from 58°C to 68°C, followed by analysis of the melting curves of the reaction products. This method makes it possible to determine whether non-specific products are present in the mixture. Since our experiments use an intercalating dye that binds to any double-stranded DNA, the specificity of the reaction is of fundamental importance for the correct evaluation of the results. Only a single peak was found for all pair primers, indicating the homogeneity of the amplicon and the high specificity of the reaction. The optimum annealing temperatures were: *ACT11* – 62°C, *THFS* – 65°C, *TMp1* – 65°C, *Th84* – 62°C, *Th113* – 65°C, *Th95* – 62°C. The efficiency of the reaction is also an important parameter when optimizing the reaction conditions. Under ideal conditions, it is 2. In our experiments, the efficiency of reactions for primers of “housekeeping” genes varied from 1.9 to 1.95, and for primers of the thaumatin II gene, from 1.79 to 1.9, with the best result for *Th84*. In some cases, the features of the primary structure of the DNA of the studied gene, as well as the high degree of nucleotide homology with other genes, do not allow to design primers providing ideal conditions for the reaction run. Since all the selected primers were suitable for real-time PCR analysis, we used the *Th84* set for the target gene and all three pairs for the “housekeeping” genes. The geometric mean of the three selected housekeeping genes was validated as an accurate normalization factor (Vandesompele et al., 2002). The level of expression of the thaumatin gene in parental line 6 was taken as 1.

**FIGURE 7:**
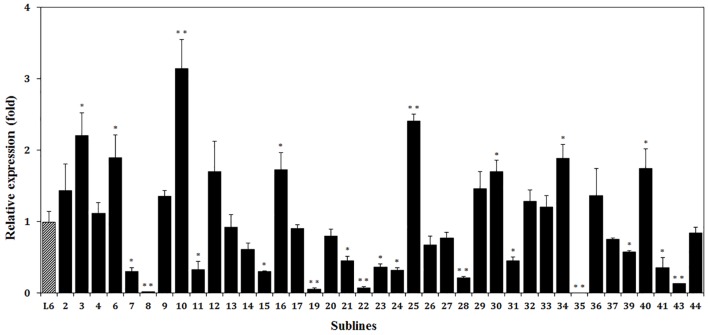
Relative expression levels of thaumatin II gene in different marker-free apple sublines. The data were normalized by combination of all reference genes. Thus, 1 is the value of parental transgenic line 6 (L6). The presented values are means of three replicates and the bars are standard deviations. ^∗^*p* < 0.05 and ^∗∗^*p* < 0.01 – significant differences by Student’s *t*-test.

Expression of the thaumatin II gene was detected in all of the marker-free sublines except subline 35 (Figure 7). The highest content of thaumatin mRNA, in transgenic apple subline 10, was three-fold higher than in the parental line. In eight apple sublines, the level of expression was statistically higher than in line 6 and in two of them it was twice as high. In nine lines, the expression of the target gene was approximately the same. In subline 35, it was not possible to detect the mRNA of the target gene. In the remaining 11 sublines, thaumatin expression was lower than in line 6. The method of quantitative evaluation of the expression of the thaumatin gene largely confirmed the results obtained with semi-quantitative RT-PCR. So, plants with a weak signal level in real-time PCR also showed extremely low values, and lines with a high signal in RT-PCR performed best in the real-time PCR analysis. Only for subline 25 the results of the two methods of analysis turned out to be different – using semi-quantitative RT-PCR failed to detect the high level of expression revealed by the real-time PCR method.

To assess the level of expression of the thaumatin II gene in marker-free plants, both methods proved to be suitable and demonstrated similar results. Semi-quantitative reverse transcription PCR allows, first of all, to exclude plants without expression, or at very low values, whereas the real-time PCR method allows a more accurate estimate to be obtained – in this case it is possible to obtain data on how much the mRNA level of the analyzed gene in a particular plant is changed with respect to other sublines and primary transgenic lines.

As a result of the analyses, we selected eight superexpressing sublines, in which the mRNA level of the thaumatin II gene exceeded the value for the parental line 6 1.5–3 times. Plants with the highest level propagated and grafted onto dwarf rootstocks for early fruit production. Our plan is to estimate the quantitative *thauII* expression of profiling in fruits next and to measure the level of protein.

## Discussion

Sweetness is a major factor in consumer preferences, and therefore, increasing sweetness is an important topic in apple breeding programs. In addition to this, research continues on the composition of apple fruits, and the effects of various metabolites on taste and sweetness (Ma et al., 2015; Aprea et al., 2017). Instead of the classical selection with its search for new wild forms with sweet fruits, a more promising approach we see is the heterologous expression of supersweet proteins in apple fruits. We have successfully worked to improve the taste of the fruits of different fruit crops, including woody plants (Dolgov et al., 2011). In the present work, we applied marker-free technologies simultaneously using only genes of plant origin to produce apple trees expressing supersweet protein. The strategy of delayed selection was used to create marker-free apple plants. This strategy involves obtaining transgenic plants and their subsequent manipulation to remove the marker gene. The working concentration of dexamethasone as recombinase activator was 10 μM in accordance with the recommendations by the system designer; evidence that the maximum induction effect is observed at Dex concentrations of 10 μM is also available (Aoyama and Chua, 1997). Vanblaere et al. (2011) use the same concentration to activate the recombinase, whereas in later work, the creators of the pMF system unexpectedly raise the concentration to 50 μM (Krens et al., 2015) which we have not compared but we achieved a higher % of rooted plants following induction with 10 μM. In another study, increasing the concentration of Dex from 10 to 50 μM did not reliably enhance (except one apple line) recombinant activity in apple and pear (Righetti et al., 2014).

In our experiments the concentration of 5-fluorocytosine was 250 mg L^-1^, which did not provide a very strong selective pressure. In most works with plants 150–300 mg L^-1^ is used (Schaart et al., 2004; Vanblaere et al., 2011; Righetti et al., 2014). Replacement of the liquid medium by a solid one at the step of cultivation with dexamethasone positively affects the viability of explants without losing efficiency (unpublished data).

We have obtained marker-free apple plants using a bifunctional selectable gene and inducible site-specific recombinase R. PCR analysis revealed that all selected plants from line 6 were free of selectable markers. The delayed selection procedure (0-2 variant) produced 11 marker-free plants and instant selection produced 13 (250-2 variant) in a first experiment. Very often delayed selection in cases with *Agrobacterium*-mediated transformation allows increasing the efficiency of regeneration, but in case of selection on 5-FC a positive effect was not observed. These similar results show that both variants are equally suitable for an effective protocol and this correlates with work in which instant selection was successfully applied to produce marker-free apple plants (Vanblaere et al., 2011; Krens et al., 2015).

Based on results obtained using Southern blotting it can be assumed that in the genome of primary transgenic line 6 there is one full copy of T-DNA and two additional inserts only with the target gene expression cassette, which remain after DNA excision. A possible tandem insertion of two copies of the target genes and a third copy into another part of the genome resulted in the loss of one of the cassettes after recombination. Additional detailed molecular investigations of the structure of inserts in marker-free plants will reliably confirm this assumption and characterize the integration and excision events. Previously, a complete molecular analysis of apple cisgenic plants carrying the scab resistance gene Rvi6 (2013) was carried out. Various combinations of multiple T-DNA inserts and the emergence of possible genotypes from a single transgenic line have been shown (Vanblaere et al., 2013). Ideally, when working with vector systems based on site-specific recombinases, one should strive to obtain transgenic plants with one whole copy of T-DNA by optimizing transformation strategies. Otherwise, in addition to the known disadvantages of multiple inserts during recombination, the likelihood of unwanted chromosomal rearrangement appears or makes DNA elimination impossible (see review Wang et al., 2011). It is thought that chromosome rearrangement or deletion one of the main negative impacts of site-specific recombinase-based systems (Yau and Stewart, 2013).

The architecture of the T-DNA copy, namely the lack of one of the RS in the genome of line 1, does not allow deleting the undesirable DNA by the prevention of site-specific recombination. We obtained five plants after the incubation of explants from line 1 (which did not contain one of the RS sites) on 5-FC, indicating that selection was insufficient. At the same time the lack (absence) of line 6 escapes leads to the conclusion that the chosen concentration of 5-FC (250 mg L^-1^) is quite adequate for routine marker-free apple plant selection. Interestingly, in early work one team of authors used a concentration of 150 mg L^-1^ (Vanblaere et al., 2011) for marker-free apple production, but in later work the concentration was increased to 500 mg L^-1^ (Krens et al., 2015).

The regeneration frequency of non-transgenic plants on medium without 5-FC and Dex was 75% in the first experiment and 45% in the second experiment. Concentrations of 5-FC ranging from 50 to 500 mg L^-1^ have been shown to be effective for negative selection at the regeneration stage (Schlaman and Hooykaas, 1997) without negative effect up to 1000 mg L^-1^ (Dai et al., 2001). Righetti et al. (2014) reported that toxicity of 5-FC measured during adventitious bud regeneration was not detectable in apple below a dose of 750 mg/l. In our hands the presence of Dex and 5-FC in the medium adversely affects non-transgenic tissue regeneration, reducing the frequency by about 1.5 times – decreasing the regeneration frequency to 55 and 27%, respectively.

Spontaneous recombination and DNA excision occurs on average in 14% of the cases (comparing variants 250-0 and 250-2) in experiments with apple tissues – in four sublines derived from explants of line 6. The observation that recombination had already taken place without treatment with Dex suggests that non-induced gene expression occurs. A similar result was obtained by Schaart et al. (2004) with strawberry plants. High level of spontaneous recombination was previously demonstrated in apple and pear: 19–34 % of the transgenic lines presented partial recombination in absence of Dex induction (Righetti et al., 2014). In our work, we used one of the well-characterized fruit-specific promoters of the tomato E8 gene to drive the expression of the thaumatin II protein gene. Although the expression of the E8 gene is controlled in both an ethylene-dependent and an ethylene-independent manner (Lincoln et al., 1987; Lincoln and Fischer, 1988; Dellapenna et al., 1989; Theologis et al., 1993) we did not find any reports regarding the expression of the E8 gene in vegetative tissues of plants. Moreover, none of the deleted forms of the promoter, not even containing an ethylene-responsive element located between -2181 and -1088, ensured the accumulation of the E8-Tag gene product in unripe fruits (Deikman et al., 1992). In another work 1.1 kb E8 gene promoter provided expression of S-adenosylmethionine hydrolase at three stages of fruit development (breaker, orange and ripe) whereas immature and mature leaves, flowers and stems, were negative for the presence of SAMase RNA (Good et al., 1994). Interestingly, that Kurokawa et al. (2013) mention in the title of their work that “…E8 promoter–HSP terminator cassette promotes the high-level accumulation of recombinant protein *predominantly* in transgenic tomato fruits…,” is apparently due to the fact that low accumulation of miraculin was detected in the green fruits. It was concluded that heat shock terminator is a strong expression enhancer that can disrupt the tissue-specificity of expression of E8 gene promoter (Kurokawa et al., 2013). However, it was previously shown that a 2181 bp version, in contrast to the deleted ones, drives the accumulation of E8 in the unripe fruits of tomato (Deikman et al., 1992). Studies on the molecular evolution of the E8 gene promoter of tomato and the search for differences between functional regions in Solanum species may allow answering emerging questions (Zhao et al., 2009). In our hands, even in tomato plants, gene expression is observed under the control of the 1193 bp promoter in vegetative tissues (Timerbaev et al., 2014). Expression levels in leaves and fruits vary considerably, but correlate with each other by result of Western blotting analysis. Quantification by ELISA showed that the protein levels of thaumatin in fruits on average 4-fold higher than the leaves (unpublished data). Due to this, it became possible to estimate the transcription level of the target gene in leaves of marker-free apple plants using semi-quantitative reverse transcription PCR and real-time PCR. We expect to obtain higher levels of expression of the *thauII* gene in apple fruits as E8 is predominantly expressed in green and ripe fruits than in leaves.

The results of apple plant selection and the analysis of T-DNA gene expression are summarized in Table 5. Altogether, 45 sublines were obtained; five of them were escapes, 40 were fully marker-free. Using real-time PCR, expression was shown in 39 marker-free plants, eight of which were selected for the rapid production of fruits and further analyses. Grafted onto dwarf rootstocks, after fruit production marker-free apple plants will be checked for taste improvement and used for future selection. So, using a vector based on the pMF system we developed an acceptable protocol for the production of marker-free apple plants – a high efficiency of excision of undesirable DNA from the apple genome was demonstrated – obtained from only one parental transgenic line. Our studies show how the scope for further improving the traits of traditional apple cultivars can be widened using marker-free technologies. The germplasm of apple tree can be expanded by genetic engineering without using genes from other taxa.

**Table 5:** Analyzed apple sublines.

Parental transgenic line	Total number of obtained sublines	Sublines expressing *nptII*/*codA* gene	Marker-free sublines	Sublines expressing *thauII* gene
1	5	5	0	5
6	40	0	40	39


## Data Availability

The datasets generated for this study are available on request to the corresponding author.

## Author Contributions

VT constructed the transformation vector, obtained marker-free apple plants, carried out PCR and qRT-PCR, and wrote the manuscript. TM obtained the transgenic apple plants. AP did the Southern blot analysis. SD acted as scientific adviser of this project and performed of plant grafting.

## Conflict of Interest Statement

The authors declare that the research was conducted in the absence of any commercial or financial relationships that could be construed as a potential conflict of interest.

## Footnotes

1 http://biochemlabsolutions.com
